# Identification of High-Yielding Genotypes of Barley in the Warm Regions of Iran

**DOI:** 10.3390/plants12223837

**Published:** 2023-11-13

**Authors:** Alireza Pour-Aboughadareh, Shirali Koohkan, Hassan Zali, Akbar Marzooghian, Ahmad Gholipour, Masoome Kheirgo, Ali Barati, Jan Bocianowski, Alireza Askari-Kelestani

**Affiliations:** 1 Seed and Plant Improvement Institute, Agricultural Research, Education and Extension Organization (AREEO), Karaj P.O. Box 31587-77871, Iran; barati32@yahoo.com; 2 Crop and Horticultural Science Research Department, Sistan Agricultural and Natural Resources Research and Education Center, Agricultural Research, Education and Extension Organization (AREEO), Zabol P.O. Box 98616-44534, Iran; koohkan182@gmail.com; 3 Crop and Horticultural Science Research Department, Fars Agricultural and Natural Resources Research and Education Center, Agricultural Research, Education and Extension Organization (AREEO), Darab P.O. Box 71558-63511, Iran; hzali90@yahoo.com (H.Z.); ar.askary@areeo.ac.ir (A.A.-K.); 4 Crop and Horticultural Science Research Department, Khuzestan Agricultural and Natural Resources Research and Education Center, Agricultural Research, Education and Extension Organization (AREEO), Ahvaz P.O. Box 61335-3341, Iran; a.marzooghian@areeo.ac.ir; 5 Crop and Horticultural Science Research Department, Golestan Agricultural and Natural Resources Research and Education Center, Agricultural Research, Education and Extension Organization (AREEO), Gonbad P.O. Box 49156-77555, Iran; ahmadgholipour@yahoo.com (A.G.); mkheirgoo@gmail.com (M.K.); 6 Department of Mathematical and Statistical Methods, Poznan University of Life Sciences, Wojska Polskiego 28, 60-637 Poznan, Poland; jan.bocianowski@up.poznan.pl

**Keywords:** multi-trait selection index, BLUP-based index, GGE biplot, AMMI analysis

## Abstract

One of the most important effects of climatic changes is increasing temperatures and expanding water deficit stress in tropical and subtropical regions. As the fourth most important cereal crop, barley (*Hordeum vulgare* L.) is crucial for food and feed security, as well as for a sustainable agricultural system. The present study investigates 56 promising barley genotypes, along with four local varieties (Norooz, Oxin, Golchin, and Negin) in four locations to identify high-yielding and adapted genotypes in the warm climate of Iran. Genotypes were tested in an alpha lattice design with six blocks, which were repeated three times. Traits measured were the number of days to heading and maturity, plant height, thousand kernels weight, and grain yield. A combined analysis of variance showed the significant effects of genotypes (G), environments (E), and their interaction (GEI) on all measured traits. Application of the additive main-effect and multiplicative interaction (AMMI) model to the grain yield data showed that GEI was divided into three significant components (IPCAs), and each accounted for 50.93%, 30.60%, and 18.47%, respectively. Two selection indices [Smith–Hazel (SH) and multiple trait selection index (MTSI)] identified G18, G24, G29, and G57 as desirable genotypes at the four test locations. Using several BLUP-based indices, such as the harmonic mean of genotypic values (HMGV), the relative performance of genotypic values (RPGV), and the harmonic mean of the relative performance of genotypic values (HMRPGV), genotypes G6, G11, G22, G24, G29, G38, G52, and G57 were identified as superior genotypes. The application of GGE analysis identified G6, G24, G29, G52, and G57 as the high-yielding and most stable genotypes. Considering all statistical models, genotypes G24, G29, and G57 can be used, as they are well-adapted to the test locations in warm regions of Iran.

## 1. Introduction

Barley (*Hordeum vulgare* L.) is an ancient and important cereal crop. It ranks fourth among all cereal crops produced in the world today, behind wheat, rice, and maize [1]. Along with wheat, barley was one of the first domesticated agricultural crops, dating from about 10,000 years ago in the Fertile Crescent in the Middle East [2]. Among cereal crops, barley in particular is genetically very diverse. It can be classified as a winter or spring, hulled or hulless grain [3], six-row or two-row, and malting or feed by end-use type. Based on grain composition, it is further classified as normal, waxy, high amylose starch types, high β-glucan, high lysine, and proanthocyanidin-free [2]. In addition, it has been shown that barley grains can be considered to be a wholesome food commodity, as they provide various minerals, phosphorus and calcium; a moderate amount of protein and fiber; and a small amount of some types of B vitamin [4]. Barley is known as a significant multi-use cereal crop, grown on more than 500 million hectares worldwide for grain, feed, fodder, and straw. It is not highly selective for soils and climate, and can therefore be grown in infertile soils and in a wide range of climates [5]. In addition, barley is one of the most well-adapted and significant cereal crops in Iran. It is also one of the key forage crops grown in different regions of the country. However, accounting for the differential response of genotypes to environmental conditions, especially climatic factors, is one of the key challenges for breeders and farmers [6].

By analyzing the genotype–environment interaction (GEI), new varieties adapted to multiple environments can be identified [7,8]. For quantitative variables such as yield, a strong GEI significantly limits the ability to select superior genotypes, as it can limit the accuracy of conclusions that would otherwise hold true [9,10,11,12,13]. Indeed, the GEI effect reduces the correlation between genotypic and phenotypic values, and hinders genetic progress in plant breeding programs. Therefore, minimizing the GEI effects is one of the main goals of any breeding program [14]. The degree of GEI can be analyzed using various graphical and numerical approaches. Among the statistical approaches, the additive main effects and multiplicative interaction (AMMI) model, and the genotype (G) main effect plus GEI (GGE) models are of great interest, as these models help breeders understand GEI patterns and evaluate the performance of genotypes under different environmental conditions. Indeed, these models allow breeders to select stable and adaptable genotypes for many environments [7,15]. The use of AMMI and GGE models has several advantages over other approaches. For example, using the AMMI model, phenotypic variation can be estimated easily and separately by separating it into the main effects of genotypes (G) and environments (E), as well as their interactions (GEI). Moreover, the model provides a path to predictive accuracy through a family member model. Furthermore, the model gives breeders the opportunity to use its valuable results of agricultural recommendations to exploit both general and specific adaptations to increase productivity [16]. The GGE biplot model, which provides graphical images of the data, is very useful for GEI interpretation. Using this model, breeders can select a high-performing genotype in a target production environment, as well as identify the target environment for a specific genotype, determine the discriminatory ability and representativeness of environments for further multi-environment trials (METs), unravel correlations between test environments, and identify ideal genotypes in terms of both yield performance and stability [17].

Genetic improvement is accelerating with the study of diverse genetic materials in many environments. Since grain yield is quantitatively inherited and depends mainly on genotypic and environmental factors, indirect selection through other agronomic traits can be useful in identifying superior genotypes [18]. Accordingly, several selection indices have been proposed based on various plant features, such as the selection index for ideal genotype (SIIG) [18], the Smith–Hazel index [19,20], the multiple trait selection index (MTSI) [21], the genotype–ideotype distance index (MGIDI) [22], and the FAI-BLUP index [23] for selecting ideal genotypes based on traits measured by multiple methods in the MET. Several studies have reported the successful use of these indices in selecting desirable genotypes [18,24,25,26,27,28]. Therefore, the use of these indicators, along with stability analysis methods, makes it possible to improve the selection process in breeding programs. In Iran, the Seed and Plant Improvement Institute (SPII) has initiated various breeding programs over the past few decades to improve and develop new cultivars for cultivation in different climates in the country. However, the implementation of newly introduced genotypes into new target environments requires a basic understanding of their yield performance, and the identification of the most suitable environments for future research and characterization of barley cultivars. In this study, we tested a set of new barley breeding genotypes that were selected from the preliminary regional barley yield trial (PBYT). Therefore, the objectives of this study were to determine the magnitude of GEI and identify ideal genotypes with high grain yield in a multi-location experiment.

## 2. Results

### 2.1. Analysis of Variance and Genetic Parameters

The results of analysis of variance showed the significant effects of genotypes (G), environments (E), and genotype by environment interaction (GEI) in terms of grain yield and other agronomic traits in the four test environments (Table 1). Of the traits measured, grain yield showed the highest phenotypic coefficient of variance (CVp) values (GY: 11.87%), followed by plant height (PLH: 5.75%), thousand kernels weight (TKW: 4.37%), the number of days to maturity (DMA: 3.23%), and the number of days to heading (DHE: 1.49%). On the other hand, GY, followed by PLH, TKW, DHE, and DMA, showed the highest genotypic coefficient of variance (CVg), (3.98, 3.28, 2.52, 1.23, and 1.18, respectively). Moreover, the GEI variance was high for all measured traits except for MDA. The broad-sense heritability (h^2^) values ranged from 0.10 (for GY) to 0.17 (for DHE). However, heritability based on the mean (h^2^_gm_) showed the highest values and ranged from 0.22 to 0.57. Of the traits measured, GY and DHE showed the lowest and highest values for this genetic parameter. The correlation between genotype and environment (R_GE_) ranged from 0.12 to 0.53, with the highest values recorded for GY, TKW, and DHE, respectively. In addition, selection accuracy (Acc) for all measured traits was high, and ranged from 0.47 (for GY) to 0.76 (for DHE). 

### 2.2. Identification of Best Genotypes Using Selection Indices

**Figure 1:**
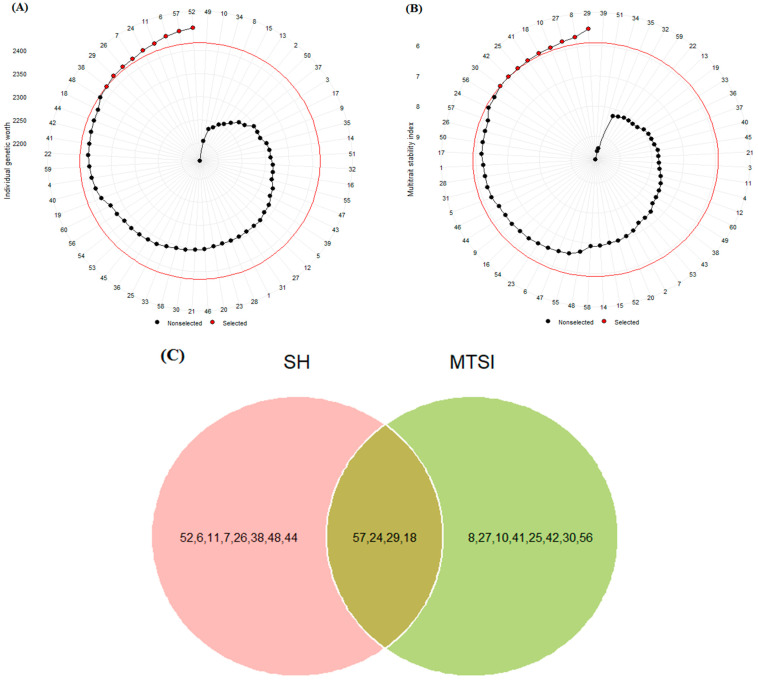
Selected barley genotypes using Smith–Hazel (**A**) and multiple trait selection (**B**) indices. The red circle represents the point separating the desired genotypes, which is marked with a red point. Venn diagram (**C**) for selected genotypes based on both SH and MTSI indices. The numbers indicate the genotype codes.

The mean values of grain yield and other measured traits at the four test locations are shown in Table 2. GY ranged from 3545.80 kg ha^−1^ to 5434.90 kg ha^−1^. PLH ranged from 77 to 94.17 cm, while TKW ranged from 36.12 g to 41.71 g. DHE showed a low index of variation and ranged from 100 to 106.50 days, while DMA showed a high index of variation and ranged from 127.17 to 143.58 days. The Smith–Hazel index (SH) and multiple trait selection index (MTSI) were used to select desirable genotypes in terms of grain yield and other agronomic traits. According to the results, SH values ranged from 2161 to 2449. As shown in Figure 1A, genotypes G2, G57 [Oxin: reference genotype], G6, G11, G24, G7, G26, G29, and G38 were identified as the best genotypes compared to other genotypes. The MTSI values for the test genotypes ranged from 5.44 to 9.77, and genotypes G29, G8, G27, G10, G18, G41, G25, G42, and G30 were identified as the best genotypes (Figure 1B). We used a Venn diagram to compare the results obtained by these indices. As shown in Figure 1C, we found that G18, G24, G29, and G57 could be selected as desirable barley genotypes based on grain yield and other agronomic traits.

### 2.3. BLUP-Based Adaptability and Stability Indices

The results of the estimated BLUP-based adaptability and stability indices, along with the average grain yield for each genotype tested, are shown in Table 2. The harmonic mean of genotypic values (HMGV) index selects genotypes with stable yield under the mixed-effect model. Among the genotypes tested, genotypes G6, G11, G22, G24, G29, G38, G41, G52, G48, and G57 [Oxin: reference genotype] with the highest values were identified as the most stable genotypes compared to other genotypes. The relative performance of genotypic values (RPGV) describes the point of adaptation. Indeed, this indicator is valuable for identifying the specific adaptability of genotypes, and can take advantage of the response of genotypes to improved growing conditions. As shown in Table 2, genotypes G52, followed by G11, G6, G24, G57 [Oxin: reference genotype], G26, G29, G38, G7, and G22 showed the highest values and were selected as adaptive genotypes. The harmonic mean of the relative performance of genotypic values (HMRPGV) has the advantage of showing the randomness of genotypic effects plus GEI effects, and provides a viewpoint of ranking patterns for genotypes according to their performance based on genetic effects. In other words, this index simultaneously shows the adaptability and stability of genotypes. Based on the HMRPGV index, the top 10 ranked genotypes are G52, G6, G24, G57 [Oxin: reference genotype], G11, G29, G38, G48, G18, and G22, respectively.

### 2.4. AMMI

The result of the AMMI analysis for the grain yield data showed that the effects of G, E, and GEI were highly significant (Table 3). The main effect of E, G, and GEI accounted for 50.22%, 9.87%, and 23.02% of the total variation, respectively. The GEI effect was divided into three significant IPCAs, each of which accounted for 50.93%, 30.60%, and 18.47%, respectively. The first four genotypes recommended for each test location were identified using the AMMI2 model. As shown in Table 3, genotype G4, G60 [Nobahar: reference genotype], G59 [Norooz: reference genotype], and G52 were identified as the four highest ranked in the Zabol location; genotypes G26, G22, G38, and G6 was observed as the highest ranking in the Gonbad location; genotypes G11, G43, G7, and G52 were dominant in the Ahvaz location; and genotypes G25, G7, G26, and G57 [Oxin: reference genotype] ranked first in the Darab location.

### 2.5. GGE Biplot Analysis

**Figure 2:**
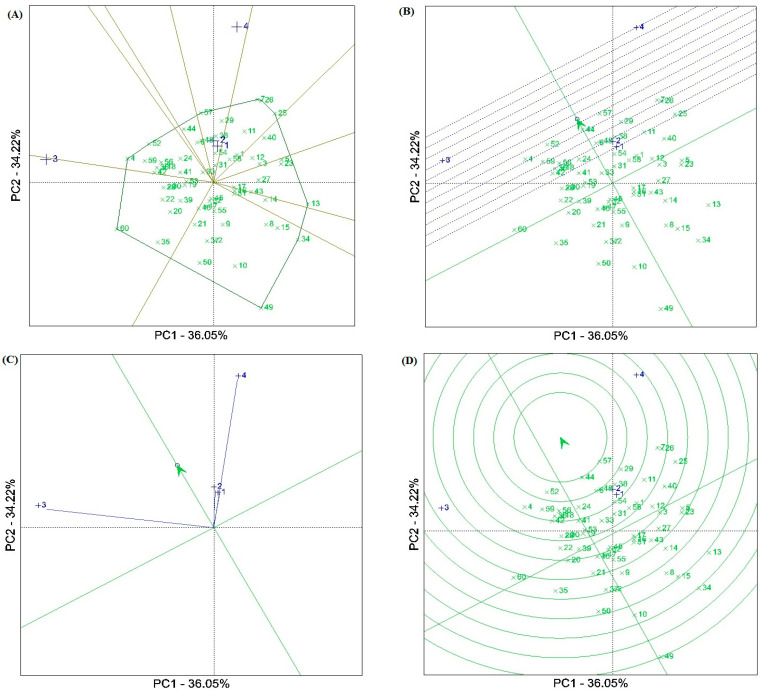
(**A**) View of the GGE ‘which–won–where’ biplot of winning genotypes for grain yield in each sector. (**B**) Biplot for simultaneous selection of grain yield and stability of barley genotypes tested. (**C**) A view of the ‘discriminating power and representativeness’ of the GGE biplot. (**D**) Comparison of promising barley genotypes with the ‘ideal’ genotype in terms of grain yield and stability at four test locations. Numbers indicade the genotype codes.

The results of the GGE biplot methodology showed that the first two components (PCAs) explained 70.26% of the total GY variation at the four test locations. The GGE biplot separated the test locations into two of the ten sectors (Figure 2A). The Ahvaz (E1), Gonbadm (E2), and Darab (E4) locations were placed in one sector, and the apex genotype for this sector was G57 [Oxin: reference genotype]. The Zabol (E3) location was placed in another sector with G60 [Nobahar: reference genotype] as the apex genotype. The “mean vs. stability” biplot viewpoint showed that genotypes G57 [Oxin: reference genotype], G44, G52, and G29 had the highest average grain yield in the locations tested. Genotype G44 showed the grain yield closest to the average value due to its position in the biplot. Genotypes G52 and G57 with a high average grain yield were the most stable, while G4, G7, and G26 showed significant yield variability across locations. However, some genotypes, such as G6, G24, G33, G32, and G45 with low average grain yield indicated high stability (Figure 2B). Figure 2C indicates the representativeness and discriminatory power of the locations. Based on this biplot, the Zabol (E3) and Darab (E4) test locations with the long environment vectors showed the highest discrimination power values. Moreover, the representative ability of the test locations was investigated using the angle between the test location vectors and the AEC (average environment coordinate). Accordingly, the Ahvaz (E1) and Gonbad (E2) locations with smaller angles indicated relatively weak representativeness, while E3 and E4 showed relatively strong representativeness. A comparative view of the GGE biplot was used to select the ideal genotypes (Figure 2D). Among the genotypes tested, G44, followed by G57 [Oxin: reference genotype], G52, G29, G38, G48, G6, G24, G18, G36, G56, G59 [Norooz: reference genotype], and G4 were near the average environment axis (AEA) and were selected as ideal genotypes. Of these, genotypes G29, G38, G48, and G6 showed specific adaptability to the E1 and E2 locations.

## 3. Discussion

In recent decades, climatic changes have affected a significant portion of agricultural fields, and agricultural products have been drastically reduced by various environmental stresses [29]. One of the most important effects of climatic changes is increasing temperatures and expanding drought stress in tropical and subtropical regions [5]. Under such circumstances, screening plant genetic materials, with an emphasis on developing high-yielding and stable varieties for cultivation in different target environments, is one of the most important breeding tasks in the world. Consequently, the development of superior genotypes requires an understanding of the GEI pattern in studies conducted at multiple locations. Progress in breeding programs to develop new varieties often depends on the performance of the genotypes tested during breeding cycles. Since grain yield is a quantitative trait, it is usually influenced by genotype, environment, and other growth traits; hence, selecting better genotypes through indirect selection using other traits can increase genetic progress [18]. Previously, most breeders applied classic stability models to identify a stable genotype [25]. These models are often determined only by grain yield data, and ignore other agronomic traits. To solve this challenge, several multi-trait-based selection indices have been proposed. One of these indices is the multiple trait selection index (MTSI) [21].

In this study, we examined a set of barley genotypes in four target locations in warm regions of Iran. Our results indicated the significant effects of genotypes (G), environments (E), and their interaction (GEI) for all measured traits. Among the sources of variation, the effects of E and GEI showed the highest total sum of squares (TSS), indicating significant differences in the genotypic response of barley to environmental conditions in the warm regions of Iran. In line with our results, previous reports also showed that the two mentioned effects were the main sources of variation in barley and other crops under different environmental conditions [30,31,32,33,34,35,36,37,38,39,40]. Among the measured traits, grain yield showed the highest phenotypic variability, while this trait showed the lowest heritability at the four test locations (Table 1). In addition, the results of AMMI analysis showed significant effects for G, E, and GEI on grain yield (Table 3). To identify desirable genotypes, two multi-trait selection indices were used, such as Smith–Hazel and MTSI. In these indices, different traits will directly play a significant role in the selection of genotypes [18]. Since the present study was conducted in warm regions, dwarfing, early earing, and maturity were taken into account as selection criteria for choosing desirable genotypes. On the other hand, the highest values of thousand grain weight and grain yield traits were taken into account in selecting desirable genotypes. Our results showed that each selection index identified different genotypes (Figure 1A,B). However, the four genotypes G18, G24, G29, and G57 were highlighted as desirable genotypes in terms of grain yield and other agronomic traits (Figure 1C). Similarly, Pour-Aboughadareh and Poczai [26,27], Selami et al. [41], Costa et al. [42], Hussain [43], and Zali et al. [18] confirmed the effectiveness of these selection indices in various crops such as wheat, lentil, mango, chickpea, and barley, respectively.

In the METs, breeders commonly use various statistical models to recommend genotypes for the target environments. One of the most important models is the best linear unbiased prediction (BLUP). This model determines the breeding values of genotypes evaluated in multiple environments, and can be used in the selection process. Moreover, the model provides a way to compare genotypes evaluated in different environments [44]. In this regard, several BLUP-based indices have been proposed to compare the genotypes evaluated in MTE experiments. One of these indicators is the harmonic mean of genotypic values (HMGV). According to Borges et al. [45], this index adjusts the predicted genetic values for the genotypes under evaluation and penalizes them based on their instability in the target environments. Relative yield of genotypic values (RPGV) is another BLUP-based index that can be used to identify the specific adaptability of each genotype, due to its ability to exploit the genotypic response to their improvements in the growing environment [46]. The third BLUP-based index is the harmonic mean of the relative performance of genotypic values (HMRPGV). This index is based on the genetic values predicted by the BLUP model, and combines estimates of adaptability and stability. Hence, HMPRGV can provides a ranking pattern for evaluated genotypes in different test environments [45]. With this in mind, the highest values of these indices can identify desirable genotypes with high stability and adaptability in MET experiments. In this regard, our results showed that genotypes G6, G11, G22, G24, G29, G38, G52, and G57 [Oxin: reference genotype] were selected as desirable compared to other genotypes.

The results of the GGE biplot analysis showed that three test locations, including Ahvaz, Darab, and Gonbad were placed in the same sector, and genotype G57 was identified as the best genotype in the sector (Figure 2A). Moreover, this genotype along with G44, G52, and G29 showed the highest yield and yield stability compared to the other genotypes (Figure 2B). A comparative view of the GGE biplot revealed that G44, followed by G57 [Oxin: reference genotype], G52, G29, G38, G48, G6, G24, G18, G36, G56, G59 [Norooz: reference genotype], and G4 were near the average environment axis (AEA) and were selected as ideal genotypes. From the viewpoint of representativeness and discrimination power of the test locations, the Zabol (E3) and Darab (E4) locations with long environmental vectors and Ahvaz (E1) and Gonbad (E2) with smaller angles on the AEC vector (average environment coordinate) showed the highest values of discrimination power and representativeness ability, respectively (Figure 2C). Hence, according to Yan’s theory [47], the Zabol and Darab locations can be classified as a Type III environment, as they are useful for eliminating unstable genotypes in breeding programs. On the other hand, although Ahvaz and Gonbad showed a short vector, they showed small angles with the AEC vector. Therefore, more information on these locations is needed to provide analysis of their role in breeding programs.

## 4. Materials and Methods

### 4.1. Plant Materials and Experiment Layouts

A set of 56 promising barley genotypes, along with four introduced local varieties (Norooz, Oxin, Golchin, and Nobahar) used as reference genotypes were tested through trials at multiple locations. All genetic materials were obtained from hybridization between national and international parents. More information on their pedigrees is shown in Supplementary Table S1. Field experiments were carried out at four warm weather test stations (including Ahvaz [31°19′13″ N, 48°40′09″ E], Darab [28°45′07″ N, 54°32′40″ E], Zabol [31°01′43″ N, 61°30′04″ E], and Gonbad [37°15′00″ N, 55°10′02″ E) in Iran during the growing seasons (2022–2023). Of these, Gonbad has warm and humid conditions and is located in the northern parts of the country, while other locations have warm and dry conditions and are located in the southern parts of Iran. In all test environments, field layouts were carried out using an alpha lattice design with three replications and six blocks. The experimental plots consisted of 6 rows, each 5 m long, with 15 cm spacing between rows. The sowing density in each plot was 300 seeds per m^2^. An experimental planter (Wintersteiger, Ried, Austria) was used for sowing. At each location, basic fertilizers such as P_2_O_5_ and N were applied at 100 and 32 kg ha^−1^, respectively, before sowing. During the growing season, five irrigations were applied at growth stages 00, 32, 51, 75, and 85 Zadoks’ [48] in all test environments. At the ZGS 31 stage, 40 kg ha^−1^ N was again applied to improve stem elongation. Based on the time of physiological maturity in each test environment, a combine harvester (Wintersteiger, Ried, Austria) was used to harvest the experimental plots. Grain yield was determined for each genotype in the test environment. Other agronomic traits measured were the number of days to heading (DHE) and maturity (DMA), plant height (PLH), and thousand kernels weight (TKW).

### 4.2. Data Analysis

The experimental data collected from the four locations were subjected to analysis of variance based on the following model using META-R software [49]:

Yijk=μ+Loci+Repj(Loci)+Blockk(LociRepj)+Genl+(Loci×Genl)+εijkl

where, Y_ijk_ is the trait of interest, μ is the overall mean effect, Loc_i_ is the effect of the location, Rep_j_ is the effect of the *j*th replicate, Rep_j_(Loc_i_) is the effect of the *j*th replication within the *i*th location, Block_k_ is the effect of the *k*th block, Gen_l_ is the effect of *l*th genotype, and ε_ijk_ is the effect of the error. 

Several genetic parameters were estimated, such as the phenotypic and genotypic coefficient of variation in percent, broad-sense heritability and heritability on the mean basis, correlation between genotype and environment, and selection accuracy for all measured traits. To select desirable genotypes based on all measured traits, two multi-traits selection indices, such as the Smith–Hazel (SH) index and multiple trait selection index (MTSI) were calculated using R software ver. 4.3.1. Moreover, several BLUP-based indices were calculated to examine the adaptability and stability of the genotypes studied, as follows:

$${HMGV}_{i}=\frac{E}{\sum_{j=1}^{E}\frac{1}{G_{vij}}}$$

$${RPGV}_{i}=\frac{1}{E}\sum_{j=1}^{E}G_{vij}/\mu_{i}$$

$${HMRPGV}_{i}=\frac{E}{\sum_{j=1}^{E}\frac{1}{G_{vij}/\mu_{i}}}$$

where, E is the number of environments and *G_vij_* is the genotypic value (BLUP) for the *i*th genotype in the *j*th environment. 

All these analyses were calculated using the ‘metan’ package [50] in R software [51]. To analyze the effect of GEI on grain yield data, AMMI analysis and GGE biplot analysis were performed. The biplots were rendered based on the first two principal components (PCA1 and PCA2). These analyses were computed using GenStat softwares ver. 23 [VSN International 2023].

## 5. Conclusions

In this study, we used several biometric models to select undesirable barley genotypes, analyze the impact of GEI, and identify high-performing genotypes in four warm regions of Iran. Ultimately, the results of this study suggest the potential of using the models as valuable tools in barley breeding programs. To conclude, our results identified genotypes G24, G29, and G57 as the superior genotypes based on all models. These genotypes can be used as well-adapted to the test locations in warm regions of Iran. Therefore, comprehensive research on these genotypes is needed before their commercial introduction.

## Figures and Tables

**Table 1:** Results of two-way analysis of variance, along with the estimated genetic parameters for measured traits.

Statistic	DHE	DMA	PLH	TKW	GY
Environment (E) significance	1.58 × 10^−8^	6.18 × 10^−9^	3.88 × 10^−5^	1.91 × 10^−5^	3.51 × 10^−3^
Genotype (G) significance	9.27 × 10^−6^	2.05 × 10^−4^	3.44 × 10^−5^	1.35 × 10^−3^	1.92 × 10^−1^
G × E significance	7.93 × 10^−9^	3.50 × 10^−3^	5.51 × 10^−16^	2.62 × 10^−9^	7.00 × 10^−11^
Genotype variance	2.71	2.72	7.67	1.69	38,262.90
G × E variance	4.12	2.93	14.39	3.88	374,092.44
Residual variance	2.56	20.60	24.71	3.05	320,923.89
Grand mean	107.36	140.31	86.52	39.97	4774.01
CVg	1.23	1.18	3.28	2.52	3.98
CVp	9.36	26.20	54.90	7.43	77.22
h^2^	0.17	0.11	0.14	0.13	0.10
h^2^_mg_	0.57	0.52	0.55	0.47	0.22
R_GE_	0.43	0.12	0.33	0.47	0.53
Acc	0.76	0.72	0.74	0.68	0.47

DHE, DMA, PLH, TKW, and GY indicate the number of days to heading, number of days to maturity, plant height (cm), thousand kernels weight (g), and grain yield (Kg ha^−1^), respectively. CVg, CVp, h^2^, h^2^_mg_, R_GE_, and Acc indicate genotypic coefficient of variance, phenotypic coefficient of variance, broad-sense heritability, heritability on the mean basis, correlation between genotype and environment, and accuracy of selection, respectively.

**Table 2:** Mean values of measured traits across four test locations, along with estimated values of selection and BLUP-based indices.

Code	DHE	DMA	PLH	TKW	GY	HMGV	RPGV	HMRPGV	SH	MTSI
G1	102.67	139.58	82.58	40.32	4753.90	4484.00	0.98	0.98	2345.00	6.24
G2	103.58	141.58	85.25	38.42	4233.80	4192.00	0.90	0.90	2266.00	7.14
G3	105.25	141.75	84.92	39.84	4444.70	4149.00	0.92	0.91	2298.00	7.77
G4	100.00	127.17	80.25	39.74	5072.20	4580.00	1.04	1.01	2394.00	7.63
G5	104.08	139.33	87.42	40.79	4701.10	4511.00	0.98	0.97	2337.00	6.27
G6	102.75	139.50	83.17	39.83	5371.20	5153.00	1.11	1.11	2439.00	6.49
G7	104.75	141.08	79.67	39.32	5266.80	4908.00	1.08	1.06	2423.00	7.27
G8	101.42	138.67	84.08	36.29	4051.70	3972.00	0.87	0.86	2238.00	5.67
G9	105.08	140.17	89.08	36.12	4448.20	4407.00	0.95	0.94	2299.00	6.39
G10	102.67	139.17	87.33	38.51	3829.30	3826.00	0.83	0.83	2204.00	5.81
G11	102.58	140.17	90.33	38.62	5328.10	5108.00	1.11	1.09	2433.00	7.73
G12	105.08	141.92	87.00	37.80	4736.00	4521.00	0.98	0.98	2342.00	7.56
G13	105.92	141.67	88.00	38.22	4165.40	4062.00	0.89	0.87	2256.00	8.11
G14	104.58	141.00	81.75	40.83	4530.80	4446.00	0.96	0.95	2311.00	6.92
G15	103.75	140.83	90.00	36.62	4124.20	4108.00	0.89	0.88	2249.00	6.97
G16	100.25	137.83	86.33	38.39	4574.40	4427.00	0.96	0.96	2318.00	6.42
G17	103.33	139.50	87.17	40.08	4447.90	4251.00	0.93	0.93	2299.00	6.18
G18	103.83	138.92	88.00	38.22	5140.30	4899.00	1.06	1.06	2404.00	5.83
G19	104.92	141.50	91.08	38.21	5020.20	4866.00	1.05	1.05	2386.00	8.07
G20	104.67	141.50	92.08	37.46	4775.10	4640.00	1.01	0.99	2348.00	7.05
G21	105.50	141.75	93.25	39.03	4806.70	4760.00	1.03	1.01	2353.00	7.78
G22	105.50	142.42	89.92	40.18	5104.40	4933.00	1.07	1.06	2399.00	8.12
G23	101.92	140.33	87.75	38.93	4755.80	4584.00	1.00	0.98	2345.00	6.47
G24	104.67	139.42	87.75	38.98	5300.20	5109.00	1.10	1.10	2428.00	5.99
G25	101.75	139.17	84.83	38.82	4869.60	4475.00	0.99	0.98	2363.00	5.89
G26	102.83	139.92	87.50	39.72	5259.20	4831.00	1.08	1.05	2422.00	6.17
G27	101.75	138.92	77.00	38.56	4737.00	4599.00	1.00	0.98	2343.00	5.73
G28	101.58	138.42	81.58	38.64	4755.50	4457.00	0.98	0.97	2345.00	6.25
G29	100.75	137.50	84.17	39.19	5252.30	4947.00	1.08	1.07	2421.00	5.44
G30	103.00	139.25	80.00	38.02	4816.70	4568.00	1.00	0.99	2355.00	5.90
G31	103.42	140.17	83.75	37.95	4739.20	4479.00	0.98	0.97	2345.00	6.25
G32	105.58	141.75	83.58	36.49	4566.70	4400.00	0.96	0.95	2317.00	8.19
G33	105.08	143.08	87.92	36.87	4840.20	4554.00	1.01	0.99	2358.00	7.95
G34	105.67	143.58	87.00	37.49	4010.00	4028.00	0.87	0.86	2232.00	9.40
G35	105.25	142.42	80.83	37.81	4507.70	4384.00	0.95	0.94	2308.00	8.22
G36	105.58	142.25	84.17	36.55	4875.00	4476.00	1.00	0.98	2364.00	7.89
G37	105.50	142.00	88.08	37.53	4346.50	4308.00	0.93	0.92	2283.00	7.88
G38	105.25	143.17	86.33	36.94	5221.50	4940.00	1.08	1.07	2416.00	7.46
G39	106.50	143.17	79.75	37.08	4696.40	4492.00	0.98	0.97	2336.00	9.77
G40	102.83	141.92	90.00	39.74	5053.10	4819.00	1.05	1.04	2391.00	7.85
G41	101.17	137.92	90.00	40.93	5105.40	4918.00	1.06	1.06	2399.00	5.86
G42	104.08	138.50	87.50	36.37	5117.50	4842.00	1.06	1.05	2401.00	5.90
G43	103.42	139.83	94.17	37.73	4644.80	4509.00	0.98	0.96	2329.00	7.32
G44	102.08	139.33	88.42	39.95	5126.70	4721.00	1.05	1.04	2402.00	6.38
G45	105.50	143.17	90.00	37.37	4883.60	4810.00	1.03	1.03	2365.00	7.78
G46	102.00	140.42	81.92	37.29	4797.90	4718.00	1.02	1.01	2352.00	6.35
G47	104.67	140.08	83.00	36.37	4636.60	4481.00	0.97	0.97	2327.00	6.53
G48	103.58	139.92	93.25	39.78	5201.50	4919.00	1.07	1.07	2413.00	6.68
G49	105.17	140.42	90.75	39.32	3545.80	3697.00	0.79	0.79	2161.00	7.49
G50	103.92	139.00	88.83	39.47	4320.20	4248.00	0.93	0.91	2279.00	6.18
G51	106.08	142.67	89.83	39.62	4534.10	4408.00	0.95	0.95	2312.00	9.79
G52	104.75	140.00	91.25	40.30	5434.90	5127.00	1.12	1.11	2449.00	6.99
G53	101.67	139.50	84.83	39.14	4897.90	4710.00	1.02	1.02	2367.00	7.29
G54	103.92	140.50	86.00	39.26	4898.80	4634.00	1.01	1.01	2367.00	6.42
G55	105.25	141.33	90.42	37.16	4581.20	4505.00	0.97	0.97	2319.00	6.55
G56	103.33	138.92	88.58	37.53	4925.30	4494.00	1.01	0.99	2371.00	5.98
G57	101.67	140.92	87.50	39.98	5407.10	5079.00	1.10	1.10	2445.00	6.15
G58	101.08	140.25	88.67	36.60	4825.20	4593.00	1.00	1.00	2356.00	6.89
G59	106.25	141.00	82.00	41.71	5089.80	4719.00	1.04	1.03	2396.00	8.12
G60	105.75	141.17	83.75	41.17	4938.50	4728.00	1.04	1.01	2373.00	7.52
±SD	1.68	2.27	3.82	1.41	402.82					

DHE, DMA, PLH, TKW, GY, HMGV, RPGV, HMRPGV, SH, and MTSI indicate the number of days to heading, the number of days to maturity, plant height (cm), thousand kernels weight (g), grain yield (kg ha^−1^), harmonic mean of genotypic values, relative performance of genotypic values, and harmonic mean of relative performance of genotypic values, respectively. SD is the standard division.

**Table 3:** The results of AMMI analysis for grain yield data.

Source of Variation		df	Sum of Square	Mean Square	*F*-Value	Variability Explained (%)	
Total		719	1.16 × 10^9^	1.62 × 10^6^			
Treatments		239	9.67 × 10^8^	4.05 × 10^7^	11.65 **		
Genotypes		59	1.15 × 10^8^	1.95 × 10^6^	5.61 **	9.87%	
Environments		3	5.84 × 10^8^	1.95 × 10^8^	48.01 **	50.22%	
Block		8	3.25 × 10^7^	4.06 × 10^6^	11.68 **		
Interactions		177	2.68 × 10^8^	1.51 × 10^6^	4.36 **	23.02%	
	IPCA1	61	1.36 × 10^8^	2.24 × 10^6^	6.44 **	50.93%	
	IPCA2	59	8.20 × 10^7^	1.39 × 10^6^	4.00 **	30.60%	
	IPCA3	57	4.95 × 10^7^	8.68 × 10^6^	2.50 **	18.47%	
Error		472	1.64 × 10^8^	3.47 × 10^5^			
**First four AMMI selections per location**							
**Location**		**Mean**	**Score**	**1**	**2**	**3**	**4**
Zabol (E3)		4951	66.92	G4	G60	G59	G52
Gonbad (E2)		4116	−10.94	G26	G22	G38	G6
Ahvaz (E1)		3859	−10.96	G11	G43	G7	G52
Darab (E4)		6169	−45.01	G25	G7	G26	G57

** Significant at *p* < 0.01.

## Data Availability

The data in this manuscript are available from the corresponding authors upon reasonable request.

## Supplementary Materials

The following supporting information can be downloaded at: https://www.mdpi.com/article/10.3390/plants12223837/s1, Table S1: The pedigree of the 56 promising genotypes of barley, along with four reference genotypes across four locations in the warm climate in Iran.
