# Supplementary material for: Identification of High-Yielding Genotypes of Barley in the Warm Regions of Iran
Source: Plants (Basel). 2023 Nov 13;12(22):3837. doi: 10.3390/plants12223837 (PMC10675262; doi:10.3390/plants12223837)
Supplement: Supplementary file 1 [file plants-12-03837-s001.zip › plants-2698653-supplementary.pdf]

Table S1. The pedigree of the 56 promising genotype of barley along with four reference genotypes across four locations in the warm climate in Iran.

Code	Pedigree
G1	Bda/Rhn-03//ICB-107766/3/Yousef
G2	Triton/Yazd-5//Nik
G3	Legia//Rhn/Lignee 527/3/EDCI-7(Pamir-013/Sonata)
G4	ATACO/COMINO//ALELI/3/BICHY2000/4/ARUPO/K8755//MORA/5/Yousef
G5	L.527/MB2367//(CI7117-9/DeirAlla106)/3/Gorgan//Aths/BC/5/Arbayan/NK1272/4/Arar/3/Mari/Aths*2//M-Att-73-337-1
G6	Sahra/3/Bda/Rhn-03//ICB-107766
G7	Sahra/3/Bda/Rhn-03//ICB-107766
G8	CHENG DU 105/4/EGYPT4/TERAN78//P.STO/3/QUINA/5/ABETO//…/6/Kavir/Arinar-C4563//WI2291/3/Karoon/Sonja/4/Beecher
G9	CHENG DU 105/4/EGYPT4/TERAN78//P.STO/3/QUINA/5/ABETO//…/6/L.527/MB2367//(CI7117-9/DeirAlla106)/3/Gorgan//Aths/BC
G10	CHENG DU 105/4/EGYPT4/TERAN78//P.STO/3/QUINA/5/ABETO//…/6/Kavir/Badia/3/Torsh/9cr.279-07/Bgs/4/Karoon/Kavi
G11	CHENG DU 105/4/EGYPT4/TERAN78//P.STO/3/QUINA/5/ABETO//…/6/Schayler/3/M.Rnb86.80/NB2905//L.527/4/Rhn-03//L.527/NK1272
G12	CHENG DU 105/4/EGYPT4/TERAN78//P.STO/3/QUINA/5/ABETO//…/6/Schayler/3/M.Rnb86.80/NB2905//L.527/4/Rhn-03//L.527/NK1272
G13	CHENG DU 105/4/EGYPT4/TERAN78//P.STO/3/QUINA/5/ABETO//…/6/L.527/MB2367//(CVI7117-9/Deir Alla106)/3/Gorgan//Aths/BC
G14	CHENG DU 105/4/EGYPT4/TERAN78//P.STO/3/QUINA/5/ABETO//…/6/EDCI-7
G15	Kavir/Arinar-C4563//WI2291/3/Karoon/Sonja/4/Beecher/5/ROHO/MAZORKA//TROMPILO
G16	NT21/Arta/3/Bda/Rhn-03//ICB-107766
G17	L.527/MB2367//(CI7117-9/DeirAlla106)/3/Gorgan//Aths/BC/4/Bda/Rhn-03//ICB-107766
G18	L.527/MB2367//(CI7117-9/DeirAlla106)/3/Gorgan//Aths/BC/4/Bda/Rhn-03//ICB-107766
G19	L.527/MB2367//(CI7117-9/DeirAlla106)/3/Gorgan//Aths/BC/5/Kavir/Badia/3/Torsh/9cr.279-07/Bgs/4/Karoon/Kavi
G20	L.527/MB2367//(CI7117-9/DeirAlla106)/3/Gorgan//Aths/BC/4/Triton/Yazd-5
G21	Zahak/1-BC-80012
G22	Nimrooz/4/Anoidium/Arbayan-01/3/Lignee527/NK1272//JLB70-63
G23	Nimrooz/4/Anoidium/Arbayan-01/3/Lignee527/NK1272//JLB70-63
G24	Nimrooz/6/CHENG DU 105/4/EGYPT4/TERAN78//P.STO/3/QUINA/5/ABETO//…
G25	Yousef/4/Anoidium/Arbayan-01/3/Lignee527/NK1272//JLB70-63
G26	Yousef/4/Anoidium/Arbayan-01/3/Lignee527/NK1272//JLB70-63
G27	GLORIA-BAR/COPAL//ABN-B/3/SHYRI/4/…/5/FLORIPONDIO/ALDE/4/CEDRO//MATNAN/EH165/…
G28	Bgs/Dujia//L.1242/3/Khoram
G29	L.527/Chn-01//Alanda/3/Alanda-01*2/4/1-BC-80152
G30	Rojo/3/LB.IRAN/Una8271//Gloria"S"/Com"S"/5/FLORIPONDIO/ALDE/4/CEDRO//MATNAN/EH165/...
G31	Kavir/Badia/3/Torsh//9cr.279-07/Bgs/4/Karoon/Kavir/5/LB.IRAN/Una8271//Gloria"S"/Com"S"/6/Nik
G32	Kavir/Badia/3/Torsh//9cr.279-07/Bgs/4/Karoon/Kavir/5/LB.IRAN/Una8271//Gloria"S"/Com"S"/6/Nik
G33	Assala'S'//Avt/Aths/3/(Arinar/Aths//D529)/4/Nik/5/Yousef
G34	Nik/Bereke-54/3/Rhn-03//L.527/NK1272
G35	Behrokh/VADA//Nik

G36	Behrokh/VADA//Nik
G37	Behrokh/VADA//Nik
G38	Nosrat/Nik*2
G39	GOB/ALELI//CANELA/3/ARUPO*2/JET/4/ARUPO/K8755//MORA/5/Lignee527/NK1272//JLB70-063/3/Barjouj/…/6/Zahak
G40	GOB/ALELI//CANELA/3/ARUPO*2/JET/4/ARUPO/K8755//MORA/5/ICNB-105960/Torkman/6/Zahak
G41	Anoidium/Arbayan-01/3/Lignee527/NK1272//JLB70-63/4/Goharan/5/Zahak
G42	Lignee 527/NK1272//JLB 70-63/3/Rhn-03//Lignee527/As45
G43	Lignee 527/NK1272//JLB 70-63/3/Rhn-03//Lignee527/As45
G44	KAROON/KAVIR/4/Rhodes'S'//Tb/Chzo/3/Gloria'S'/5/Legia
G45	KAROON/KAVIR/4/Rhodes'S'//Tb/Chzo/3/Gloria'S'/5/Legia
G46	Cr115/Por//Bc/3/Api/CM67/4/Giza120/5/H272/Bgs/3/Mzq/Gva//...Alanda-01/6/Sahra
G47	Rojo/3/LB.IRAN/Una8271//Gloria"S"/Com"S"/4/Triton/Yazd-5
G48	Beecher/4/Rihane-03/3/As46/Aths*2//Aths/Lignee686
G49	Legia//Rhn/Lignee 527/3/Rhn-03//L.527/NK1272
G50	Legia//Rhn/Lignee 527/3/Rhn-03//L.527/NK1272
G51	Yousef/3/Legia//Rhn/Lignee 527
G52	KAROON/KAVIR/3/Rhodes'S'//Tb/Chzo/4/Gloria'S'/5/Nik/6/Yousef
G53	WB-99-6
G54	WB-99-10
G55	WB-99-11
G56	WB-95-9
G57	Oxin [Reference]
G58	Golchin [Reference]
G59	Norooz [Reference]
G60	Nobahar [Reference]
